# Average Power Estimation in the 15 s Repeated Jump Test: A Comparison of the Bosco, MG, and MGM-15 Models with an Exploratory BikeErg Benchmark

**DOI:** 10.3390/jfmk11040379

**Published:** 2026-09-23

**Authors:** Vlad Adrian Geantă, Pierre Joseph de Hillerin, Anca Ionescu, Alexandra Reta Iacobini

**Affiliations:** 1 Doctoral School of Sport Science and Physical Education, Pitesti University Center, National University of Science and Technology Politehnica Bucharest, 110040 Pitesti, Romania; hillerin@live.com (P.J.d.H.); anca.ionescu@unitbv.ro (A.I.); alexandra.iacobini@spiruharet.ro (A.R.I.); 2 Department of Physical Education and Sport, Faculty of Physical Education and Sport, Aurel Vlaicu University of Arad, 310025 Arad, Romania; 3 Research Center for Physical Activities, Aurel Vlaicu University of Arad, 310330 Arad, Romania; 4 Neuromotrica-Information for Sport and Human Performance Ltd., 021323 Bucharest, Romania; 5 Department of Motric Performance, Faculty of Physical Education and Sports Mountain, Transylvania University of Brasov, 600115 Brașov, Romania; 6 Department of Physical Education, Sport and Kinesitherapy, Faculty of Physical Education and Sport, Spiru Haret University, 030045 Bucharest, Romania

**Keywords:** repeated jump test, average power output, psycho-neuro-motor, vertical jump, MGM-15, Bosco, ergometer

## Abstract

**Background:** Average power in repeated vertical jump tests is commonly estimated rather than directly measured, and the equation chosen may substantially affect interpretation. This quasi-experimental within-subject study compared Bosco, MG, and MGM-15 estimates from identical 15 s repeated-jump kinematic data and, as a supplementary exploratory component, quantified their differences relative to power from a separate 20 s Concept2 BikeErg trial. **Methods:** Fifty-one physically active male participants performed a 15 s repeated vertical jump test with arm swing on OptoJump. Raw kinematic data were converted to average power using the Bosco, MG, and MGM-15 equations. Average power was also recorded during a 20 s maximal BikeErg trial. Repeated-measures ANOVA, Pearson correlations, linear regression, and Bland–Altman analyses quantified between-method and cross-task differences. **Results:** Mean power differed markedly across models: 40.79 ± 7.65 W/kg (Bosco), 21.60 ± 5.13 W/kg (MG), and 4.20 ± 0.54 W/kg (MGM-15), versus 7.83 ± 0.94 W/kg for BikeErg (F(1.03, 51.29) = 1082.05, *p* < 0.001, η^2^p = 0.956). MGM-15 correlated strongly with Bosco (r = 0.977) but produced substantially lower estimates. Bland–Altman mean differences relative to BikeErg were +32.96 W/kg (95% LoA: 18.69 to 47.22) for Bosco, +13.77 W/kg (4.34 to 23.20) for MG, and −3.63 W/kg (−5.27 to −2.00) for MGM-15; proportional bias was confirmed for all three models (all *p* < 0.001). **Conclusions:** Average power values from this test are strongly model dependent. Bosco, MG, and MGM-15 values are not interchangeable, even if they come from the same kinematic dataset. MGM-15 yielded estimates nearer to BikeErg power than either Bosco or MG. However, since cycling and jumping are biomechanically different activities performed over different time spans, this indicates only exploratory numerical convergence, not criterion validity.

## 1. Introduction

Lower-limb average power is a fundamental determinant of neuromuscular performance and a key indicator in sports performance assessment [1,2]. Vertical and repeated jump tests are widely used to assess explosive strength, stretch-shortening cycle function, fatigue resistance, and sport-specific performance under sport-relevant conditions [3,4,5]. Their widespread use in monitoring neuromuscular performance and training adaptations has established repeated jump tests as one of the most applied field-based assessments in sport science [6,7,8,9,10].

The 15 s repeated vertical jump protocol and its associated computational model, later known as Modified Miron Georgescu-15s (MGM-15), originated at the Romanian National Research Institute for Sport and was built on the multi-jump methodology introduced by Miron Georgescu [11]. During the 1970s, Pierre de Hillerin adapted this methodology into its 15 s repeated-jump form and formulated the corresponding power-estimation model [12]. The Romanian state sports system during the communist period disseminated research mainly through internal institutional reports, so the protocol and model remained largely outside the international literature. Early institutional documentation dates from 1982 [13] and the model reached the open literature only in 1997 through republished institutional material [12,14].

Without access to this earlier work, Bosco, Luhtanen, and Komi proposed an independent method for estimating power during the same 15 s protocol [4], using jump count and cumulative flight time. Owing to its earlier international publication, this approach became the most widely disseminated computational method for the protocol and was incorporated into several commercial and research systems [15,16,17,18]. These systems do not directly measure mechanical power; rather, power is estimated from flight time (T_f_) and contact time (T_c_), so the resulting value depends on the model’s mathematical assumptions as well as on actual performance [19,20,21].

This matters because different computational models applied to identical repeated-jump data can produce substantially different power estimates. Geantă and de Hillerin [22] first reported systematic differences between the Bosco, MG, and MGM-15 models, and a larger-sample study quantified an average discrepancy of approximately 36 W/kg between Bosco and MGM-15. Part of this divergence follows directly from each model’s embedded assumptions: MG applies a multiplication factor of 1.5 derived from an assumed two-phase contact model, while Bosco applies a larger factor of 2 and divides exclusively by contact time, implicitly treating muscular output as continuous across the jump cycle. MGM-15 applies no such multiplier, distributing the calculation across both contact and flight time. Such differences reflect distinct operational definitions of “mean power” rather than a single construct estimated with varying precision, and cannot, by themselves, be interpreted as differences in neuromuscular performance [23,24]. The critical question is therefore not whether models differ, but how their estimates relate to an independently recorded measure of mechanical power [25].

A preliminary exploratory study addressed this by comparing Bosco, MG, and MGM-15 estimates with power recorded on the Concept2 BikeErg and RowErg [26]. Despite the small sample (n = 5), Bosco and MG consistently exceeded both ergometers, while MGM-15 did not differ significantly from RowErg and was only slightly lower than BikeErg, suggesting closer numerical proximity to ergometer-derived power. However, the exploratory sample size precluded conclusions about criterion validity or interchangeability [27]. Such cross-task benchmarking is not unprecedented: Bosco, Luhtanen, and Komi [4] themselves compared jump-derived power with Wingate, Margaria, and sprint performance. In this work, BikeErg power likewise serves as an independent mechanical reference rather than a universal criterion. Cycling and jumping involve different movement patterns; the comparison is intended to characterize numerical bias, not interchangeability between modalities.

Unlike Bosco, which relates work primarily to contact time, MGM-15 incorporates both contact and flight time, yielding more conservative estimates [14,22]. This distinction has also been discussed within a psycho-neuro-motor framework of motor performance [14,28,29]. In the present context, this framework refers to the execution of the jump task, including upper-limb contribution, rather than to psychological or neural variables measured directly.

MGM-15 has been applied across volleyball, football, judo, basketball, gymnastics, handball, athletics, and dance [30,31,32,33,34,35,36,37,38]. Despite this longstanding use, MGM-15 has not been examined in a larger international peer-reviewed study against an independently recorded mechanical-power reference. This gap is relevant because repeated-jump metrics are increasingly used to monitor neuromuscular performance and training-related changes in applied settings [10,39,40], yet how closely their outputs track directly measured mechanical power remains uncharacterized.

Accordingly, the aim of this study was to compare average power estimates from the Bosco, MG, and MGM-15 models applied to identical kinematic data from the 15 s repeated jump test, and to quantify their paired numerical differences relative to directly recorded Concept2 BikeErg average power. Building on prior between-model comparisons and the preliminary five-participant ergometer study [26], this investigation extends the cross-task comparison to a larger, adequately powered cohort. Two hypotheses were set out. First, the three models were expected to yield markedly different power estimates from identical kinematic data because of their different mathematical formulations. Second, MGM-15 was expected to exhibit the smallest numerical deviation from BikeErg average power, as suggested by the preliminary study, a question of numerical closeness only, not of greater validity or equivalence to mechanical power.

## 2. Materials and Methods

### 2.1. Research Design

This study employed a single-group, quasi-experimental within-subject design combined with a preliminary external-reference comparison component to examine whether the MGM-15 model shows closer numerical agreement with directly measured ergometer power than the classical Bosco model in a 15 s repeated vertical jump test.

Each participant performed the same repeated jump protocol, and the resulting raw kinematic data were processed using different computational models, allowing direct comparison of model-derived power values from identical performance input. In addition, all participants completed a 20 s maximal-effort Concept2 BikeErg trial, which served as an external, kinematics-independent mechanical reference for the agreement analysis. The design was intended to evaluate both the relationship between jump-based computational models and the extent to which their outputs numerically approximated average power values recorded under standardized ergometer conditions. While sample size was determined based on the comparative (ANOVA) component of the design, the agreement analysis is hypothesis-generating in nature and intended to inform, rather than confirm, future validation studies with dedicated agreement-based sample size calculations.

### 2.2. Participants

Fifty-one physically active male university students enrolled in Physical Education and Sport-related programmes volunteered to take part in this study (age 20.9 ± 2.3 years, height 180.5 ± 6.8 cm, body mass 75.5 ± 10.0 kg). Their mean countermovement jump height was 47.4 ± 7.0 cm, indicating a generally active and sport-oriented sample.

Recruited on a voluntary basis from multiple sport disciplines without restriction to a specific athletic background, participants met predefined inclusion and exclusion criteria, as the study aimed to evaluate computational model agreement across a physically active, non-elite population rather than within a sport-specific cohort.

Inclusion criteria required participants to be male (to minimize sex-related variability in stretch-shortening cycle mechanics), physically active, engaged in organized sports training at least three times per week during the previous six months, and familiar with maximal effort in repeated vertical jump testing. This criteria was consistent with established stretch-shortening cycle research, in which sex-related differences in tendon stiffness and elastic energy return have been documented [41]. Exclusion criteria included any current or recent musculoskeletal injury, history of lower-limb surgery, or cardiovascular, neurological, or balance-related disorders that could affect maximal effort performance or compromise testing safety. Elite athletes were not included to avoid excessive heterogeneity associated with highly specialized training backgrounds.

Participants were instructed to avoid strenuous physical activity for at least 24 h before testing and to maintain their usual hydration and sleep habits. Written informed consent was obtained from all participants before data collection. The study protocol was approved by the institutional ethics committee of Aurel Vlaicu University of Arad, Faculty of Physical Education and Sport (Registration number: 312/15.06.2026), in accordance with the Declaration of Helsinki. This sample was recruited independently of, and did not overlap with, the exploratory cohort (n = 5) reported in our earlier preliminary comparison [26], which was conducted under a separate ethics approval issued in an earlier period. The flow of participant recruitment, eligibility screening, and inclusion in the final analysis is presented in Figure 1. Of the 75 individuals assessed for eligibility, 24 were excluded, including 16 who did not meet the inclusion criteria, 4 who declined to participate, and 4 excluded for other reasons. No participants withdrew during the study, and all 51 enrolled participants were included in the final analysis.

### 2.3. Procedure

All assessments were conducted indoors under standardized environmental conditions in the same testing facility. Before testing, participants received verbal instructions and a practical demonstration of each task. A standardized warm-up was then performed, consisting of 5 min of light jogging, dynamic mobility exercises, and several submaximal vertical jumps to ensure adequate neuromuscular preparation [42].

#### 2.3.1. Jump Test

After the warm-up, participants completed a single 15 s repeated vertical jump test (Figure 2) with arm swing on the OptoJump Next system [43], following previously described protocols for 15 s “like a ball” jumps [14,22]. They were instructed to jump “like a ball”, as high and as elastically as possible throughout the 15 s period, aiming to maximize flight time while minimizing ground contact time. The OptoJump system recorded flight time and contact time for each jump with a temporal resolution of 0.001 s and automatically provided average power output values based on the embedded Bosco equation [4].

The raw kinematic data (flight time, contact time, and jump height) were exported for offline analysis and subsequently used to compute additional average power estimates according to the Miron Georgescu (MG) and MGM-15 models [14,22].

#### 2.3.2. Cycling Test

Following the jump assessment, participants completed a 20 s short-duration maximal cycling test (Figure 3) on a Concept2 BikeErg (Concept II BikeErg; Concept2 Inc., US) [44], as previously described in exploratory work comparing jump-based power models with ergometer-derived power outputs [26]. The ergometer was preferred over force-platform-based assessment as an external reference because it captures whole-body external mechanical power output under standardized loading conditions, independent of the kinematic variables (flight time, contact time) embedded in the jump models under evaluation.

Cycling and jumping engage different muscle action patterns and contraction types [2,45], and direct interchangeability between cycling-based and jump-based anaerobic power assessments has been empirically questioned even when correlations between the two are statistically significant [46]. This is precisely why BikeErg was chosen: the goal was not biomechanical similarity to jumping, but a kinematics-independent benchmark against which jump-derived estimates could be tested. A force platform, though biomechanically closer to jumping [47,48], still derives power from the same flight-time and contact-time inputs used by the models under evaluation, making it unsuitable as an independent reference. Concept2 ergometers are widely used for standardized performance testing, and matched workloads on the BikeErg and RowErg have been shown to evoke systematic, interpretable physiological responses in trained athletes [27,49]. This choice of a cycling-based external reference follows an established precedent in this research area and is revisited in detail in Section 4.4, including its relationship to methodological independence and historical validation practice.

After individualized adjustment of saddle height and handlebar position, participants were instructed to accelerate as fast as possible and sustain maximal effort throughout the 20 s trial, following manufacturer and laboratory recommendations. Mean power output, as reported by the PM5 performance monitor, was recorded and normalized to body mass (W/kg).

A standardized 5 min passive recovery period separated the jump test and cycling trial, consistent with recovery durations reported after short-duration maximal efforts [50,51]. Testing order was fixed (jump test first) rather than randomized, since the two tasks share no kinematic variables; this was considered unlikely to bias jump-derived data, though the non-randomized sequence remains a limitation acknowledged in Section 4.5.

### 2.4. Power Calculation Models

Raw kinematic data exported from OptoJump—flight time (T_f_), contact time (T_c_), and jump height (h)—were used as the input for three computational models of average power output (PU): the Miron Georgescu (MG) model [11], the Bosco model [4], and the Modified Miron Georgescu-15 (MGM-15) model [14,22]. The average power models are the following:

(1) $$\mathrm{MG}\;\mathrm{Methodology}:\;\;\;\;\;\;\;\;PU=1.5\times \;\frac{g^{2}\;\times \;{T_{f}}^{2}}{8\;\times {\;T}_{c}}$$

where PU_MG_ is average power output (W/kg), g is gravitational acceleration (9.81 m/s^2^), T_f_ is flight time (s), and T_c_ is contact time (s). This method emphasizes explosive efficiency by relating squared flight time to ground contact duration [14,22].

(2) $$\mathrm{Bosco}\;\mathrm{Methodology}:\;\;\;\;\;\;\;\;PU=2\times \frac{g^{2\;}\times T_{f}\times 15}{4n\times (15-T_{f})}$$

where PU_Bosco_ is average power output (W/kg), g is gravitational acceleration (9.81 m/s^2^), *T_f_* is the total cumulative flight time across all jumps (s), n is the number of jumps completed during the trial, and 15 represents the duration of the repeated jump test (s). The original Bosco equation was developed for a 60 s protocol [4]; in the present study, the test duration constant was replaced with 15 s to match the experimental protocol, following previous adaptations [14,22].

Average power output according to the classical Bosco model was obtained directly from the OptoJump software, which implements the original Bosco methodology adapted for a 15 s repeated jump protocol [4,14,22]. In this approach, cumulative flight time and the number of jumps completed within the 15 s test are used to estimate mechanical power from repeated vertical displacements.

(3) $$\mathrm{MGM}\text{-}15\;\mathrm{Methodology}:\;\;\;\;\;\;\;\;PU=\frac{g^{2}\times {T_{f}}^{2}}{8\times \left(T_{c}+T_{f}\right)}$$

where PU_MGM-15_ is average power output (W/kg), g is gravitational acceleration, T_f_ is flight time (s), and T_c_ is contact time (s). This updated formula distributes power across both contact and flight time in the denominator, providing a more conservative and time-specific representation of lower-limb power over the full contraction–relaxation cycle [14,22].

For all participants, MGM-15 and MG calculations were performed from the raw jump-by-jump OptoJump export. Bosco values were obtained from the OptoJump output; the software configuration and calculation setting used for the 15 s protocol were recorded and retained.

### 2.5. Worked Example of PU

For reproducibility, a worked example of the MGM-15 calculation is provided using raw OptoJump kinematic data [14]. The system exports flight time (T_f_) and contact time (T_c_) for each jump, with jump height derived from flight time. In one representative jump, Tc was 0.185 s and T_f_ was 0.537 s, corresponding to a jump height of 0.353 m. Substitution of these values into Equation (3) gives:

(4) $$PU=\frac{9.81^{2}\times {\left(0.537\right)}^{2}}{8\times \left(0.185+0.537\right)}=\frac{96.24\times 0.2884}{8\times 0.722}=\frac{27.75}{5.776}=4.80\;W/kg$$

This value represents the MGM-15 power estimate for that jump. The same procedure was applied to each jump recorded during the 15 s test, and the arithmetic mean of the individual jump estimates was used as the participant-level MGM-15 value. All parameters required for the calculation are contained in the raw OptoJump XML export, allowing the procedure to be reproduced without additional data collection. The calculation follows the framework described by Geantă et al. [14].

### 2.6. Statistical Analyses

All statistical analyses were performed to examine differences, relationships, and agreement between average power values obtained from the three computational models and the BikeErg reference.

Descriptive statistics were calculated as mean ± standard deviation (M ± SD) and coefficient of variation (CV %) to characterize central tendency and dispersion for anthropometric variables and all power-related outcomes. An a priori power analysis was conducted using G*Power version 3.1.9.7 [52] for a one-way repeated-measures ANOVA with four levels assuming a medium effect size (f = 0.25), an estimated correlation among repeated measures of r = 0.5, no sphericity correction (ε = 1), an alpha level of 0.05, and desired power (1 − β) = 0.80. This analysis indicated that a minimum total sample size of 24 participants would be required. The final sample of 51 participants exceeded this minimum requirement and was therefore considered sufficient to provide adequate statistical power for detecting medium-sized effects in the main effect of method. This computation supports identifying average differences across methods, but it does not establish the precision of the Bland–Altman limits of agreement reported in Section 3.3. Accordingly, those results are regarded as hypothesis-generating rather than confirmatory.

Normality was verified using the Kolmogorov–Smirnov test, first on the four raw method-level variables (MG, Bosco, MGM-15, BikeErg), supporting the repeated-measures ANOVA used to compare methods, with Bonferroni-adjusted pairwise comparisons and effect sizes quantified as partial eta squared (η^2^p; small ≥ 0.01, medium ≥ 0.06, large ≥ 0.14) according to Cohen’s guidelines [53]. Normality was also evaluated for the paired differences between each model and BikeErg with the Kolmogorov–Smirnov test. Each of the three difference distributions (MG–BikeErg, Bosco–BikeErg, MGM-15–BikeErg) was compatible with a normal distribution (all *p* > 0.05), which justified the later Bland–Altman analyses.

Pearson correlation and linear regression between MGM-15 and Bosco were used to characterize the structural mathematical relationship between the two models, not to assess agreement, whereas the association between MGM-15 and BikeErg was examined to explore the degree of shared variance between the computational estimate and the external mechanical reference.

Agreement with BikeErg was evaluated via Bland–Altman analysis [54] for all three models (MG, Bosco, MGM-15), with MGM-15 treated as the model of primary interest and Bosco as the most widely adopted reference formula. No a priori acceptability threshold was defined, given the absence of an established biomechanical criterion for this comparison; limits of agreement are reported descriptively relative to the BikeErg mean. Proportional bias was formally tested by regressing paired differences on the corresponding mean for each model.

All statistical tests were two-tailed, and the level of significance was set at *p* < 0.05. The analyses were performed using IBM SPSS Statistics, version 31.0 (IBM Corp., Armonk, NY, USA).

## 3. Results

Descriptive statistics for all four methods are presented in Table 1. Mean power output differed substantially across methods when applied to identical kinematic data, with Bosco producing the highest estimates (40.79 ± 7.65 W/kg), approximately ten times greater than MGM-15 (4.20 ± 0.54 W/kg). BikeErg power averaged 7.83 ± 0.94 W/kg, a value closer in magnitude to MGM-15 than to any other computational model. The lowest variability was observed for BikeErg (CV = 12.02%) and MGM-15 (CV = 12.76%), suggesting comparable inter-individual dispersion between these two methods.

### 3.1. Differences Between Methods

A one-way repeated-measures ANOVA revealed a statistically significant effect of method on average power output. Mauchly’s test indicated that the assumption of sphericity was violated (Greenhouse–Geisser ε = 0.342) and degrees of freedom were consequently corrected using the Greenhouse–Geisser estimate: F(1.03, 51.29) = 1082.05, *p* < 0.001, η^2^p = 0.956 (Table 2), indicating that a substantial proportion of the within-subject variance in estimated power output was associated with the method.

Bonferroni-adjusted pairwise comparisons confirmed that all method pairs differed significantly (*p* < 0.001), as detailed in Table 3. The largest discrepancy was observed between Bosco and MGM-15 (mean difference = +36.59 W/kg, 95% CI: [33.85, 39.33]), followed by Bosco vs. BikeErg (+32.96 W/kg, 95% CI: [30.16, 35.76]). The smallest mean difference was observed between MGM-15 and BikeErg (−3.63 W/kg, 95% CI: [−3.95, −3.31]).

The magnitude of these differences is further illustrated in Figure 4, which displays mean ± SD power output for each method with annotated significance brackets for key comparisons.

### 3.2. Relationships Between Methods

Pearson correlations and linear regression coefficients are summarized in Table 4. MGM-15 demonstrated a near-perfect linear relationship with the Bosco model (*r* = 0.977, *p* < 0.001), with a regression slope of *B* = 13.95 (95% CI: [13.09, 14.82]), indicating that each 1 W/kg increase in MGM-15 was associated with an estimated 13.95 W/kg increase in Bosco-derived power. This scaling relationship reflects the structural amplification embedded in the Bosco formula rather than a difference in physical performance, as visually confirmed in Figure 5. In contrast, the association between MGM-15 and BikeErg was moderate (*r* = 0.473, *p* < 0.001), with *B* = 0.83 (95% CI: [0.39, 1.28]), indicating a moderate association between MGM-15-derived estimates and BikeErg power, as illustrated in Figure 6.

### 3.3. Agreement with BikeErg Reference Values

Bland–Altman analysis indicated that MGM-15 had a mean bias of −3.63 W/kg versus BikeErg (95% CI: [−3.87, −3.40]), equal to 46% of the BikeErg mean. The 95% limits of agreement ranged from −5.27 to −2.00 W/kg, and the LoA width was 3.27 W/kg (Figure 7). In the case of MG, the mean bias was +13.77 W/kg relative to BikeErg (95% CI: [12.42, 15.12]; 176% of the BikeErg mean), with 95% LoA from +4.34 to +23.20 W/kg and an LoA width of 18.86 W/kg (Figure 8). Bosco showed a mean bias of +32.96 W/kg (95% CI: [30.91, 35.00]; 421% of the BikeErg mean), and its 95% LoA extended from +18.69 to +47.22 W/kg, with an LoA width of 28.53 W/kg (Figure 9).

Regressing the paired differences against the mean of each model and BikeErg verified significant proportional bias in all three models (Table 5): Bosco (B = 1.75, R^2^ = 0.952, *p* < 0.001), MG (B = 1.63, R^2^ = 0.894, *p* < 0.001), and MGM-15 (B = −0.73, R^2^ = 0.313, *p* < 0.001). For every comparison, the paired differences met the normality assumption (Kolmogorov–Smirnov, all *p* > 0.05).

## 4. Discussion

This study evaluated mean power estimates from the Bosco, MG, and MGM-15 models in relation to directly obtained Concept2 BikeErg power. In this quasi-experimental design, the preliminary exploratory-reference element indicated that MGM-15 was numerically nearer to the ergometer benchmark than Bosco. Even though every method relied on the same kinematic inputs, the outputs differed markedly: Bosco yielded the greatest values (40.79 W/kg), whereas MGM-15 gave the smallest (4.20 W/kg). BikeErg power (7.83 W/kg) was closest to MGM-15, and the Bland–Altman mean bias for MGM-15 (−3.63 W/kg) was much lower than Bosco’s positive bias (+32.96 W/kg). Building on our earlier methodological series [14,22,26] these results extend it to a larger, statistically powered cohort and indicate that the observed model gaps arise from mathematical formulation rather than true performance variation.

### 4.1. Structural Basis of the Higher Bosco Power Estimates

Bosco attributes all mechanical work to contact time alone, treating the musculoskeletal system as active only during propulsion. Since the “like a ball” protocol minimizes contact time by design, dividing by this small denominator generates markedly higher numerical estimates under this 15 s jump test. The Bland–Altman plot revealed a further concern: disagreement with BikeErg increased progressively at higher power values. In other words, the estimation error increases disproportionately with jumping proficiency, particularly among individuals relying more heavily on elastic return, which may risk misattributing elastic contributions to active muscular output [41,55,56].

This structural limitation was previously demonstrated by applying the Bosco formula to a bouncing basketball, a purely elastic system with no muscular input. The formula overestimated power by roughly 365 times [28], a Popperian critical test [57] of the claim that Bosco strictly quantifies muscular power. The present dataset extends this qualitative demonstration quantitatively: the same amplification operates systematically in human data and scales with performance level.

### 4.2. Structural Basis for the Exploratory Numerical Convergence of MGM-15 with BikeErg Power

MGM-15 incorporates both contact and flight time in the denominator, distributing work across the full contraction–relaxation cycle. This structural difference explains the empirical result from our study: a small mean bias (−3.63 W/kg) and narrow limits of agreement (−5.27 to −2.00 W/kg; width 3.27 W/kg) versus BikeErg.

Correlation and agreement, however, answer different statistical questions: Pearson’s r reflects the strength of a linear association, whereas Bland–Altman analysis captures the actual numerical closeness between paired values [54]. A moderate correlation can therefore coexist with narrow limits of agreement, as observed here, when individual-level variability lowers the linear coefficient without disrupting the overall numerical proximity between methods [58]. Accordingly, the present agreement findings are best interpreted through the Bland–Altman results rather than through correlation strength alone, while the moderate r value (0.473) signals that individual-level predictive precision between the two methods remains limited and merits further investigation.

This partial dissociation between correlation and agreement is, in part, expected on conceptual grounds: MGM-15 estimates mechanical power from jump kinematics, whereas BikeErg measures external power during a distinct cycling task. Repeated-jump performance also reflects elastic energy use, stretch-shortening efficiency, and psycho-neuro-motor coordination beyond muscular output alone, so only partial correspondence between the two measures should be expected; a perfect correlation would, in fact, be biomechanically implausible.

Conceptually, jumping is a two-phase cycle: contraction cannot be dissociated from the preceding relaxation phase [22]. Within this cycle, the muscular component can be conceptualized as a “two-stroke engine”, executing a contraction stroke followed by a relaxation stroke with each jump. By anchoring the denominator to both phases, MGM-15 self-corrects for flight time, aligning estimates with a psycho-neuro-motor framework that integrates psychological, neural, and muscular contributions across the motor chain [22,28,29,59]. This pattern extends the preliminary observations of Geantă and de Hillerin [26], who compared jump-derived models against both BikeErg and RowErg in a small exploratory sample: MGM-15 showed no significant difference from RowErg and only a slight underestimation relative to BikeErg, an early, if modest, signal of convergence with directly measured mechanical output. The present study confirms and quantifies this BikeErg-specific pattern with formal bias and agreement-limit estimates in a statistically powered cohort.

Taken together, these results suggest repeated-jump performance should not be interpreted solely as anaerobic power capacity. Since the protocol maximizes height while minimizing contact, elastic energy storage and anticipatory neuromuscular control may also contribute meaningfully to ballistic performance [60,61]. However, given that this comparison involves a biomechanically distinct task (cycling) assessed over a different duration, MGM-15’s closer numerical proximity to BikeErg should be interpreted as preliminary, exploratory convergence between tasks rather than evidence that MGM-15 more validly captures elastic, anticipatory, or psycho-neuro-motor contributions to jump performance [28,29,60,61].

### 4.3. Methodological and Reporting Implications

Model choice carries real consequences, not just theoretical ones. As shown above, Bosco and MGM-15 diverge by roughly 36 W/kg on identical data, a gap large enough to compromise cross-study comparability when methods go unstandardized or unreported [19,24]. Newer approaches using signal processing and machine learning [62] point to the same conclusion: how you calculate matters as much as what you measure.

This has a direct practical consequence. OptoJump Next and similar systems output Bosco values by default. A practitioner or a coach unfamiliar with the underlying formula has no way of knowing the estimate does not reflect the athlete’s actual mechanical output. Fortunately, switching models requires no additional data collection when raw exports are available. For example, MGM-15 uses the same raw XML export, only flight and contact time, so no extra data collection is needed (see Section 2.5 for the worked calculation).

More broadly, these findings push back against treating repeated jump protocols as pure anaerobic power tests. Between the elastic energy contribution during fast stretch-shortening cycles [55], the bias that grows with jump proficiency, and MGM-15’s closer match to ergometer output, the case builds for reframing these tests as integrative psycho-neuro-motor assessments [28,29].

### 4.4. Historical Context and Previous Benchmarking

Independent benchmarking gaps are not unique to MGM-15. Bosco originally validated his test against the Wingate anaerobic test [4]. Bosco-derived values exceeded Wingate outputs, yet this gap went unquantified for four decades. Kaufmann et al. [63] later confirmed it: Bosco power exceeded Wingate by more than twofold (20.8 vs. 9.1 W/kg), reflecting different metabolic pathways. Our results echo this pattern in a new ergometer context. The gap also has a historical root. The Miron Georgescu method dates to 1953. MGM-15 followed in the 1970s. Both preceded Bosco et al. [4] by years. Communist-era publishing restrictions, not scientific delay, kept them out of the international literature until 1997. Bosco became the global standard simply by publishing first. Our findings add preliminary, exploratory support for MGM-15’s practical values, not confirmatory validation. Its closer numerical match to BikeErg power suggests relevance, but Bosco’s earlier international adoption reflects timing, not superiority.

### 4.5. Limitations

Several limitations of the present study warrant explicit acknowledgement. First, although the Concept2 BikeErg provides directly measured power output that is independent of jump kinematics, it does not constitute a biomechanically identical reference for vertical jumping. The present comparison therefore addresses mechanical proximity rather than construct equivalence, and the absence of a force-platform reference should be considered when interpreting the observed agreement. A related limitation was the duration mismatch between the 15 s repeated jump protocol and the 20 s BikeErg trial. Since average power in maximal efforts depends on the length of the measurement window, the BikeErg value may in part capture fatigue that builds up over the extra 5 s. Accordingly, it cannot be treated as a criterion value matched for duration. The 20 s duration was used because it was the minimum programmable interval available on the PM5 monitor. This mismatch may have contributed to the observed bias and should be addressed in future studies using synchronized 15 s ergometer data acquisition or a device permitting a 15 s interval. Second, the sample comprised physically active male university students recruited without restriction to a specific sport discipline, a deliberate choice to isolate the model comparison from sport-specific technical and physiological heterogeneity, though it limits generalizability to female athletes, elite athletes, sport-specific cohorts, and other age groups. A companion study in preparation applies a stratified design across four sport disciplines involving plyometric training. Future studies should replicate these analyses in sex-balanced, sport-diverse samples across performance levels. Third, all testing was conducted in a single session without a test–retest assessment, precluding conclusions about the reliability of the observed agreement across occasions. Indirect evidence from a separate repeated-measures intervention study using the same 15 s MGM-15 protocol reported good-to-excellent relative reliability across three testing sessions (ICC = 0.767–0.879; Cronbach’s α = 0.819–0.907) [64], suggesting that the protocol itself is stable across repeated administrations. Nevertheless, a dedicated test–retest reliability analysis of the present ergometer-referenced comparison remains necessary to establish the stability of the observed relationship. Fourth, the assessment sequence was predetermined instead of being counterbalanced. Even though all participants completed a standardized 5 min passive recovery interval, lingering neuromuscular fatigue could have lowered cycling power output. That possible influence is not neutral, because reduced BikeErg output would make MGM-15 and BikeErg appear numerically closer, rather than farther apart. Under the current design, this directional confounding cannot be excluded. In future work, the test order should be randomized or counterbalanced, and enough recovery time should be provided between conditions. Fifth, despite standardized instructions, inter-individual variation in jumping technique may have introduced additional variability into the kinematic data and influenced model-derived estimates differently across participants. This source of variability may be particularly relevant when comparing a kinematics-based power estimate with directly measured ergometer power. Sixth, the moderate correlation observed between MGM-15 and BikeErg (r = 0.473) indicates that the present findings should be interpreted as preliminary evidence of concurrent validity rather than as confirmatory validation. The correlation alone does not establish equivalence between the two methods, particularly in the absence of a biomechanically matched criterion measure and repeated assessments. Replication in larger and more diverse samples, incorporating repeated testing and additional external mechanical references, is therefore required before MGM-15 can be considered a fully validated alternative to the Bosco model.

### 4.6. Practical Implications

For practitioners and researchers using OptoJump or similar systems, the present findings suggest a straightforward recommendation: raw kinematic data should be exported and processed using MGM-15 alongside or in place of the default Bosco output, particularly when the objective is to obtain a power estimate that approximates externally measurable mechanical output. The computation requires no additional equipment and can be implemented directly from existing XML exports as described in the worked example in Section 2.5.

MGM-15 shows a smaller cross-sectional coefficient of variation (12.76%) than Bosco (18.75%) and MG (23.76%), indicating less dispersion between subjects at this single measurement point. Importantly, this coefficient captures inter-individual variability rather than test–retest error, and it does not show greater sensitivity to changes within an athlete; demonstrating that attribute would require a dedicated test–retest design, as stated in Section 4.5. Even so, repeated jump assessment is commonly used in applied contexts to describe performance level, including elite judo [65] and monitoring of explosive-power training [66]. That usage highlights why it is practically important to specify what cross-sectional dispersion measures can and cannot justify. In addition, the increasing adoption of cycling-based ergometers in team-sport conditioning [67] supports the practical viability of combining ergometer-derived benchmarks with jump-based monitoring in applied sport environments.

## 5. Conclusions

This study shows that mean power estimates from a 15 s repeated vertical jump test vary markedly according to the calculation equation applied. That result emerges from the quasi-experimental within-subject comparison of Bosco, MG, and MGM-15 using the same kinematic dataset, and it indicates that outputs produced by these three equations should not be treated as equivalent measures.

In the study’s preliminary exploratory reference component, MGM-15 yielded values that were numerically nearer to the mean power from a 20 s BikeErg test than did the Bosco equation. However, cycling and jumping are biomechanically distinct tasks evaluated over different time spans, and the present comparison was exploratory. The observed difference should therefore be interpreted only as an initial indication of numerical alignment across tasks rather than as evidence of criterion validity. Before any conclusions are made about the validity, accuracy, reliability, or sensitivity of MGM-15, additional research is required using protocols matched in time and direct measurements of mechanical power during the same jump test.

These findings underscore the need to report the computational equation used in repeated-jump testing with clarity. Users of OptoJump or similar systems should be cautious when interpreting default Bosco outputs and, when raw flight-time and contact-time data are accessible, may wish to compute MGM-15 as a supplementary estimate, but not as though its closeness to ergometer power already establishes validity.

This work is an early exploratory part of a wider research program focused on MGM-15. Future studies should examine test–retest reliability, responsiveness to training-related change, and concordance with complementary external mechanical reference, preferably through temporally matched protocols and direct jump-specific power measurement, and within a confirmatory rather than exploratory design, before any claims about validity, accuracy, or sensitivity are made.

## Figures and Tables

**Figure 1:**
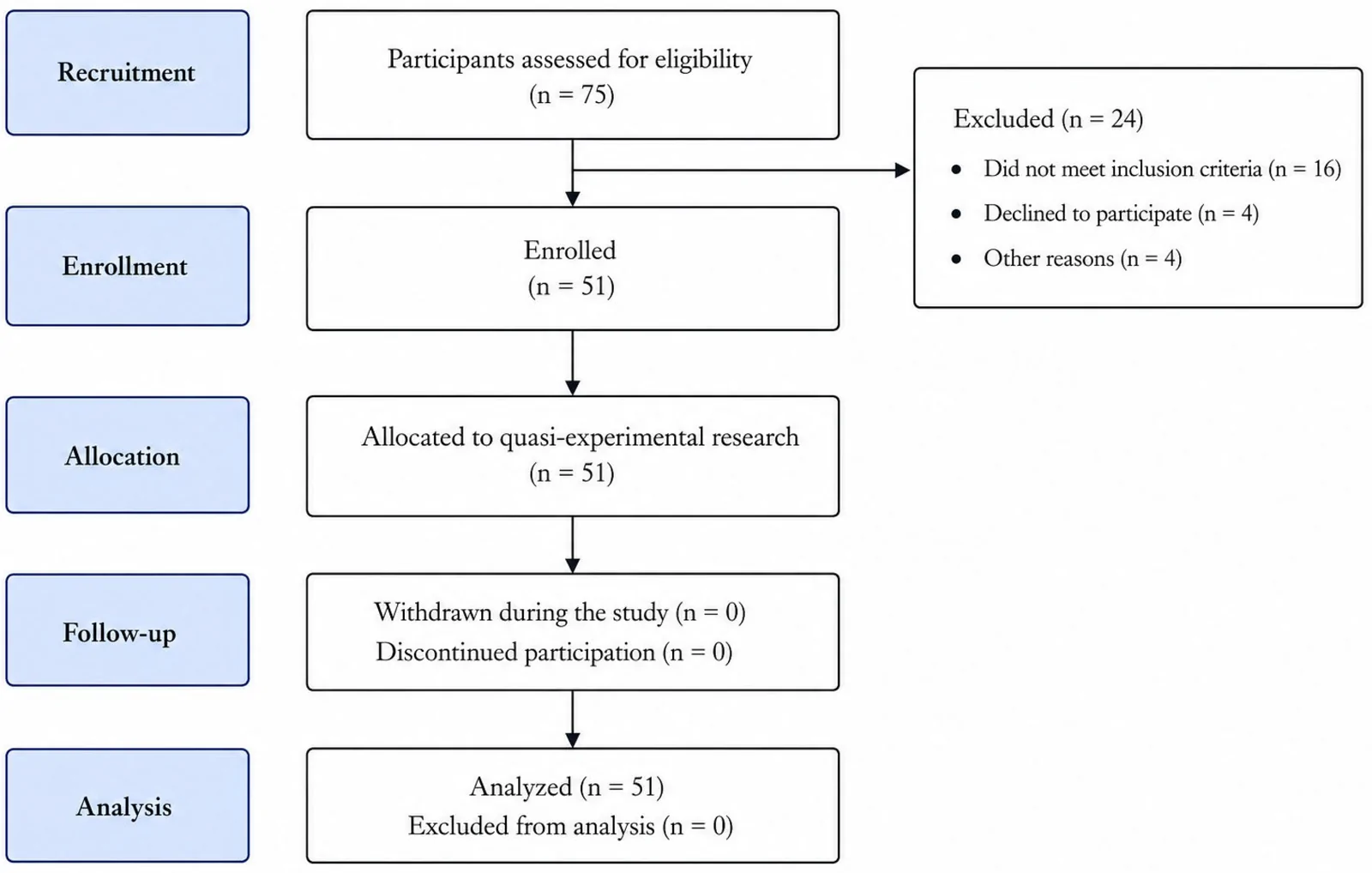
Flow diagram of participant recruitment, eligibility screening, allocation, follow-up, and inclusion in the final analysis.

**Figure 2:**
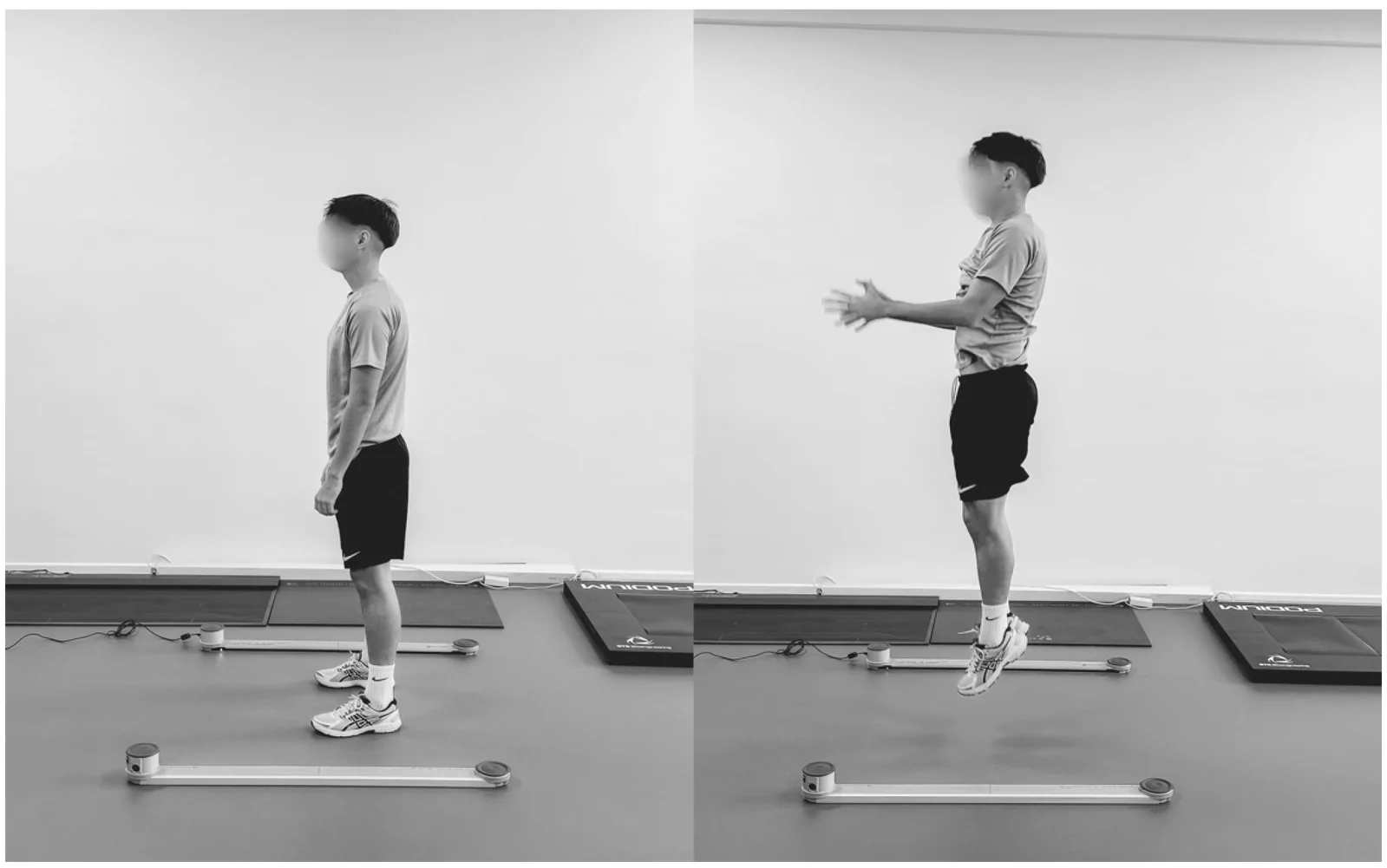
Participant performing the 15 s vertical jump test (“like a ball”) on the OptoJump Next system.

**Figure 3:**
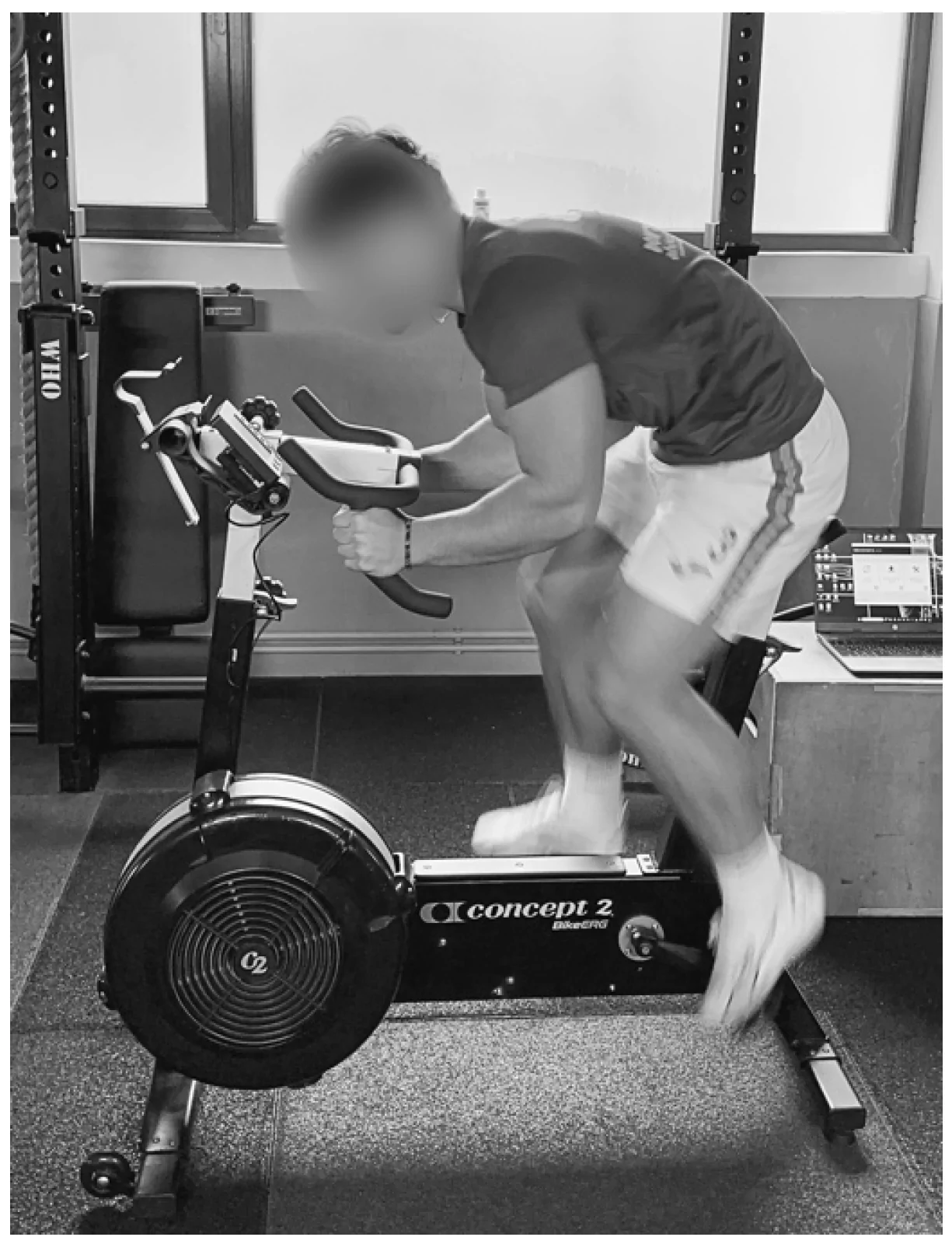
Participant performing the maximal short duration cycling test on the Concept2 BikeErg ergometer.

**Figure 4:**
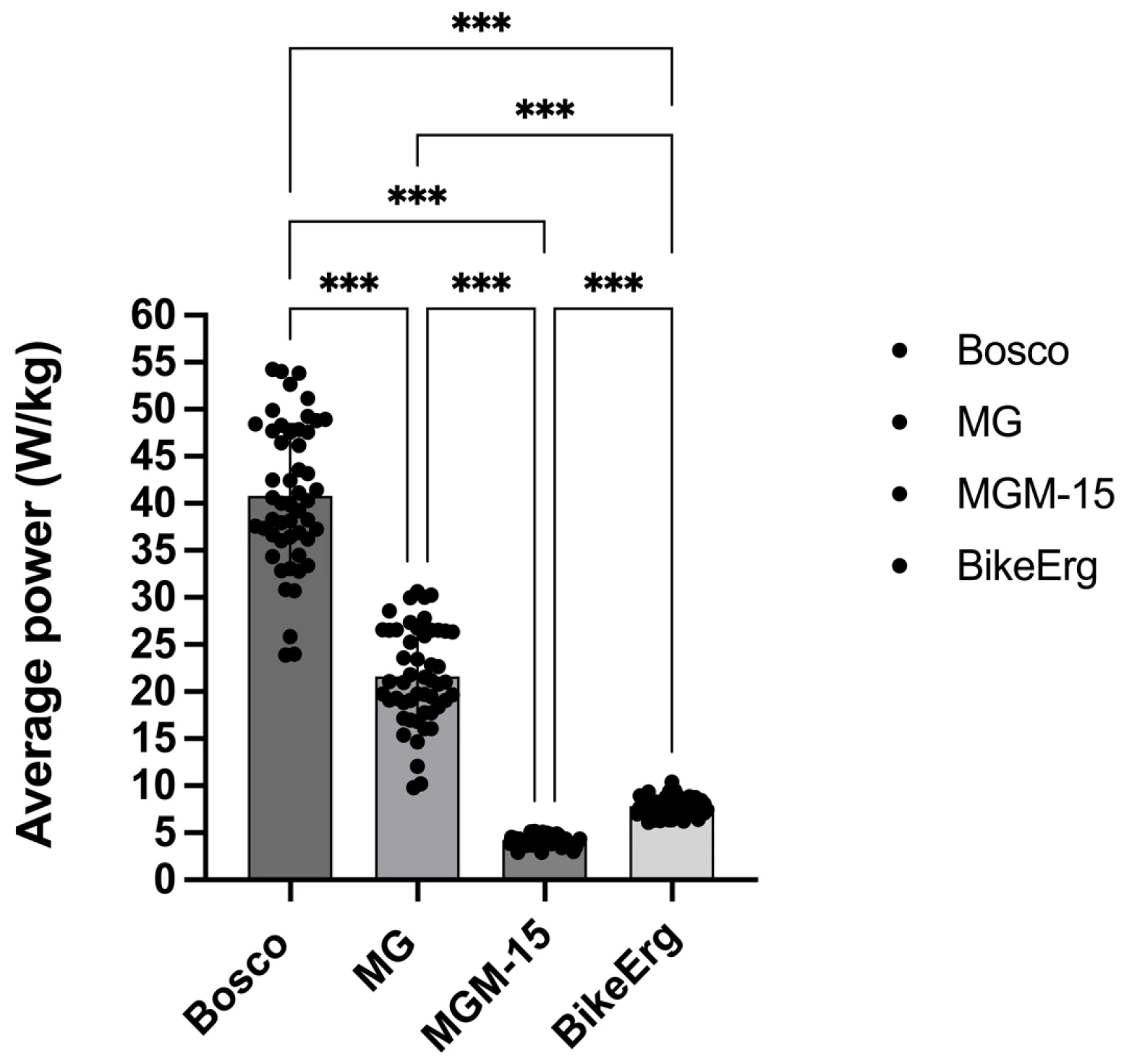
Mean ± SD power output with Bonferroni-adjusted pairwise significance brackets. *** indicates a statistically significant difference at *p* < 0.001. All comparisons: *p* < 0.001. Only key comparisons are annotated for clarity: Bosco vs. MGM-15, Bosco vs. BikeErg, and MGM-15 vs. BikeErg.

**Figure 5:**
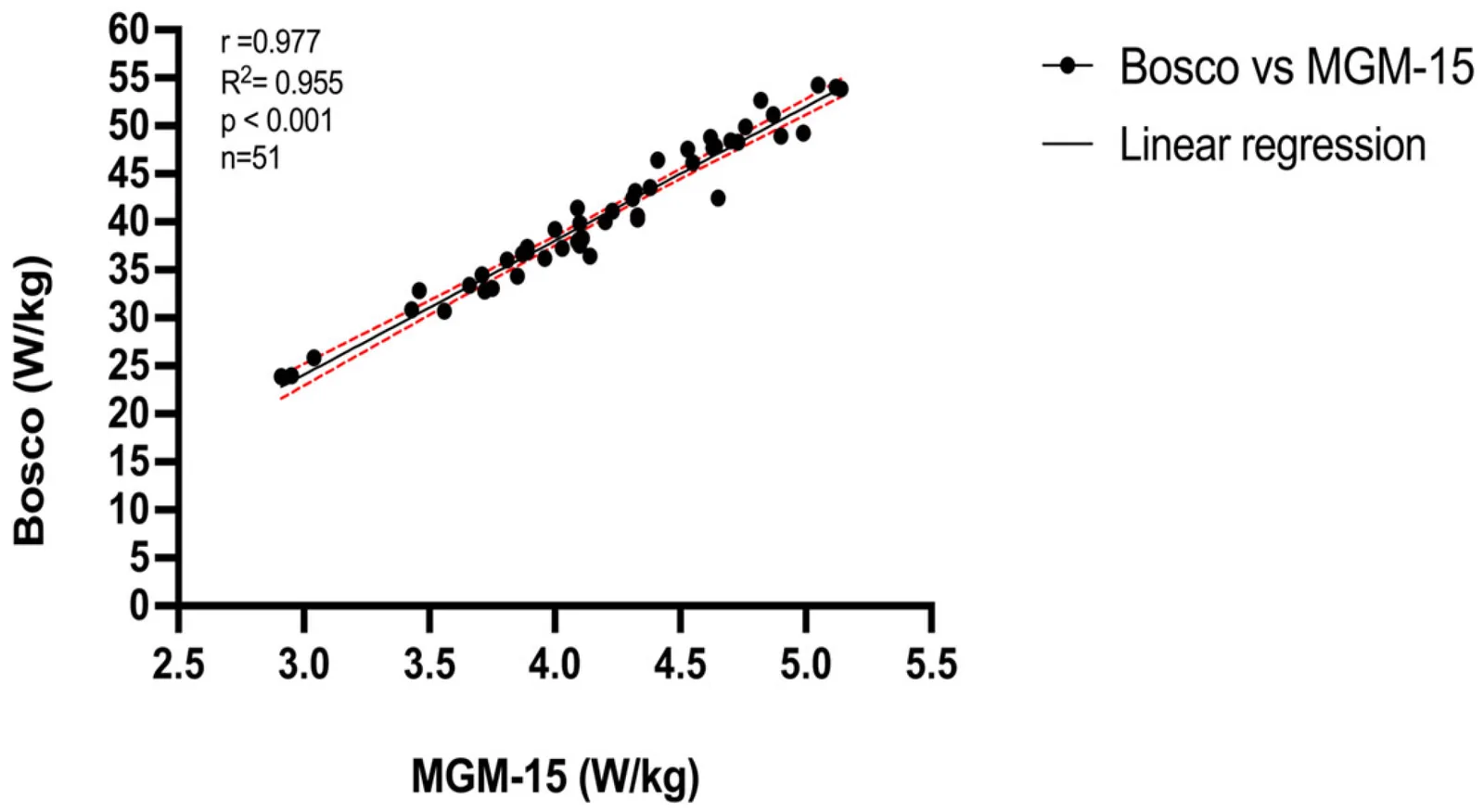
Scatterplot with a linear regression line (solid black line) and 95% confidence interval limits (red dashed lines): MGM-15 vs. Bosco.

**Figure 6:**
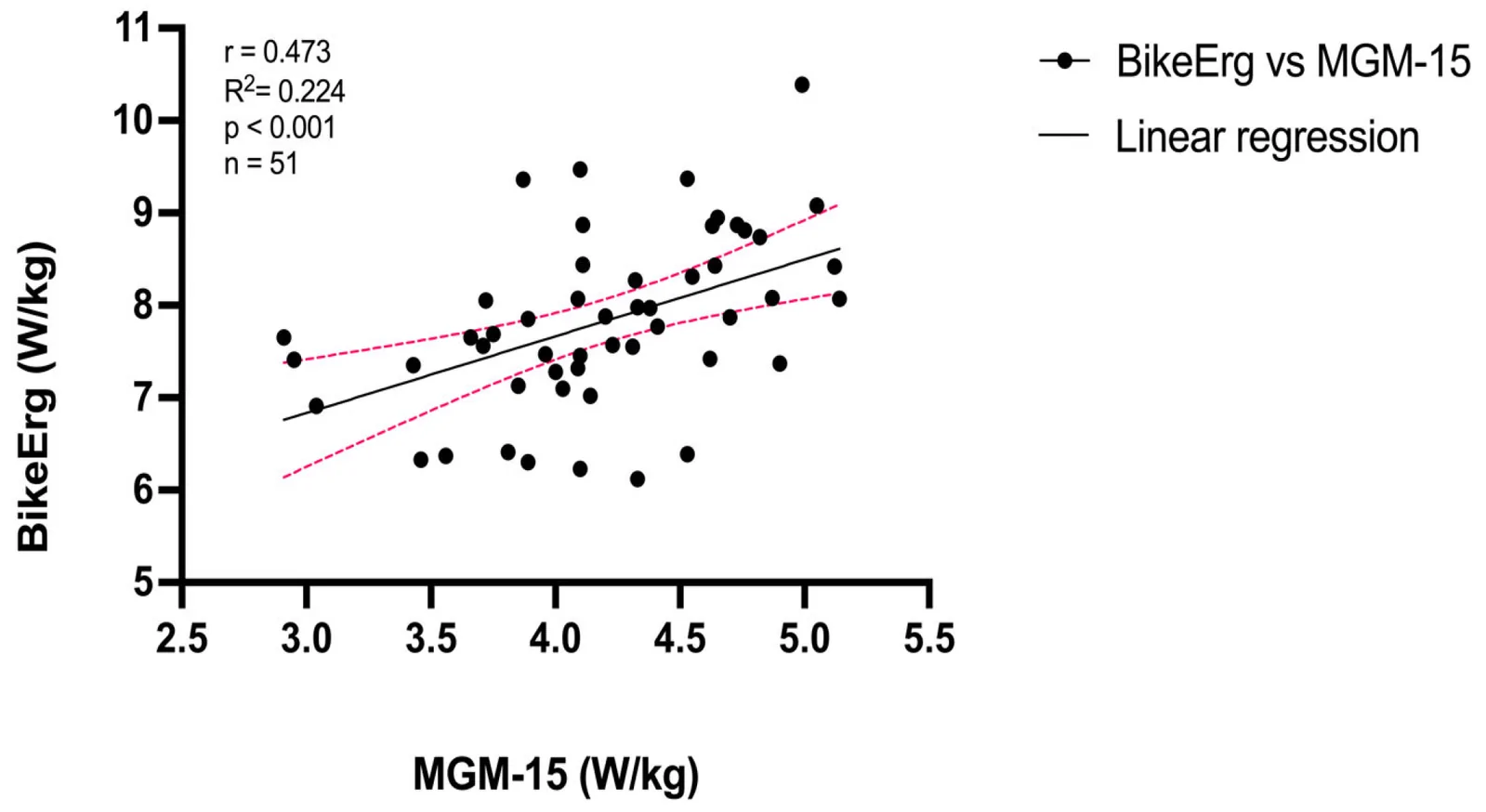
Scatterplot with a linear regression line (solid black line) and 95% confidence interval limits (red dashed lines): MGM-15 vs. BikeErg.

**Figure 7:**
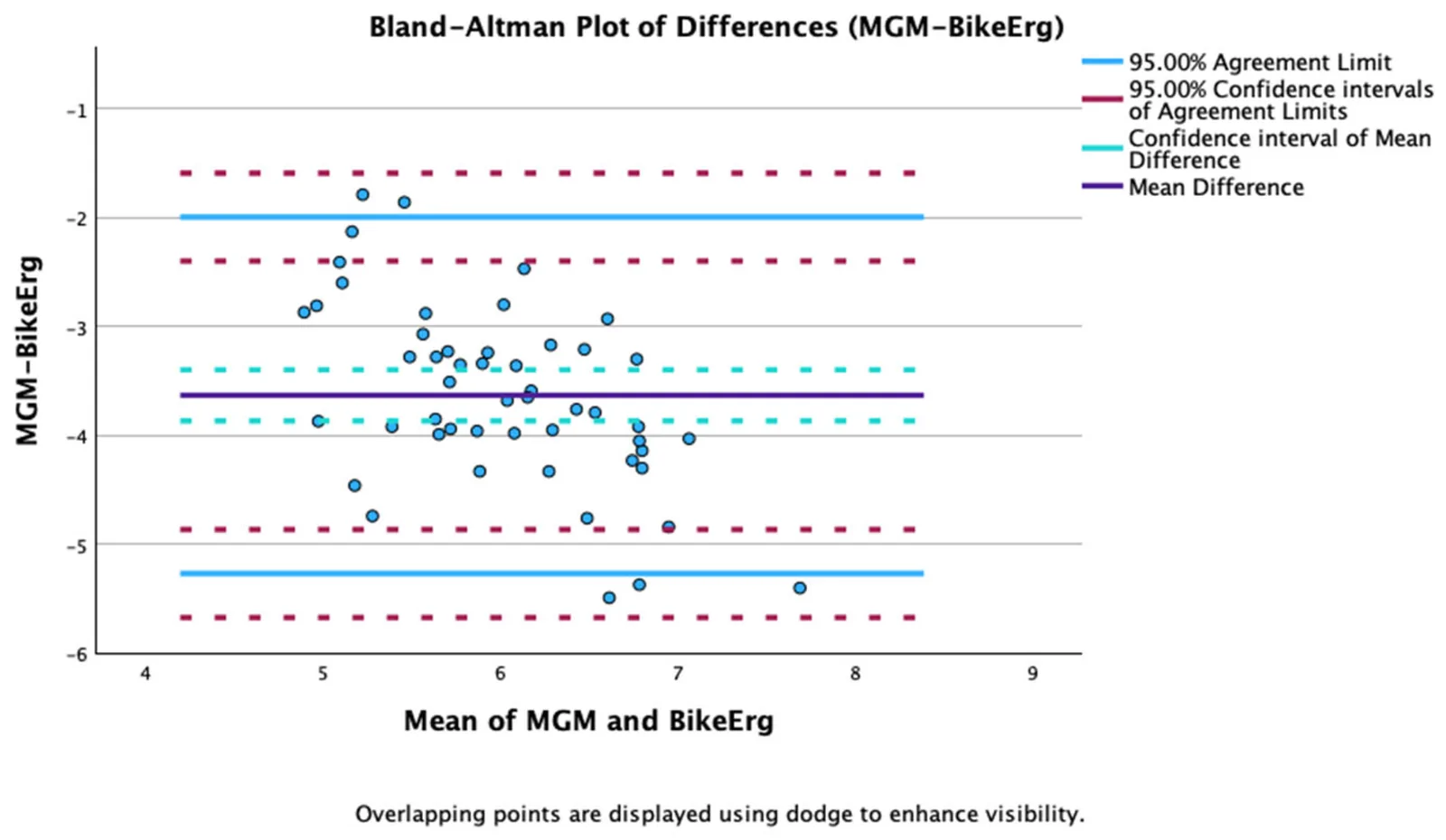
Bland–Altman plot illustrating agreement between MGM-15 estimates and BikeErg measurements. The solid grey line indicates the mean difference; the dotted turquoise lines indicate the 95% confidence interval for the mean difference. The solid blue lines indicate the 95% limits of agreement, and the dashed red lines indicate the 95% confidence intervals for the upper and lower limits of agreement.

**Figure 8:**
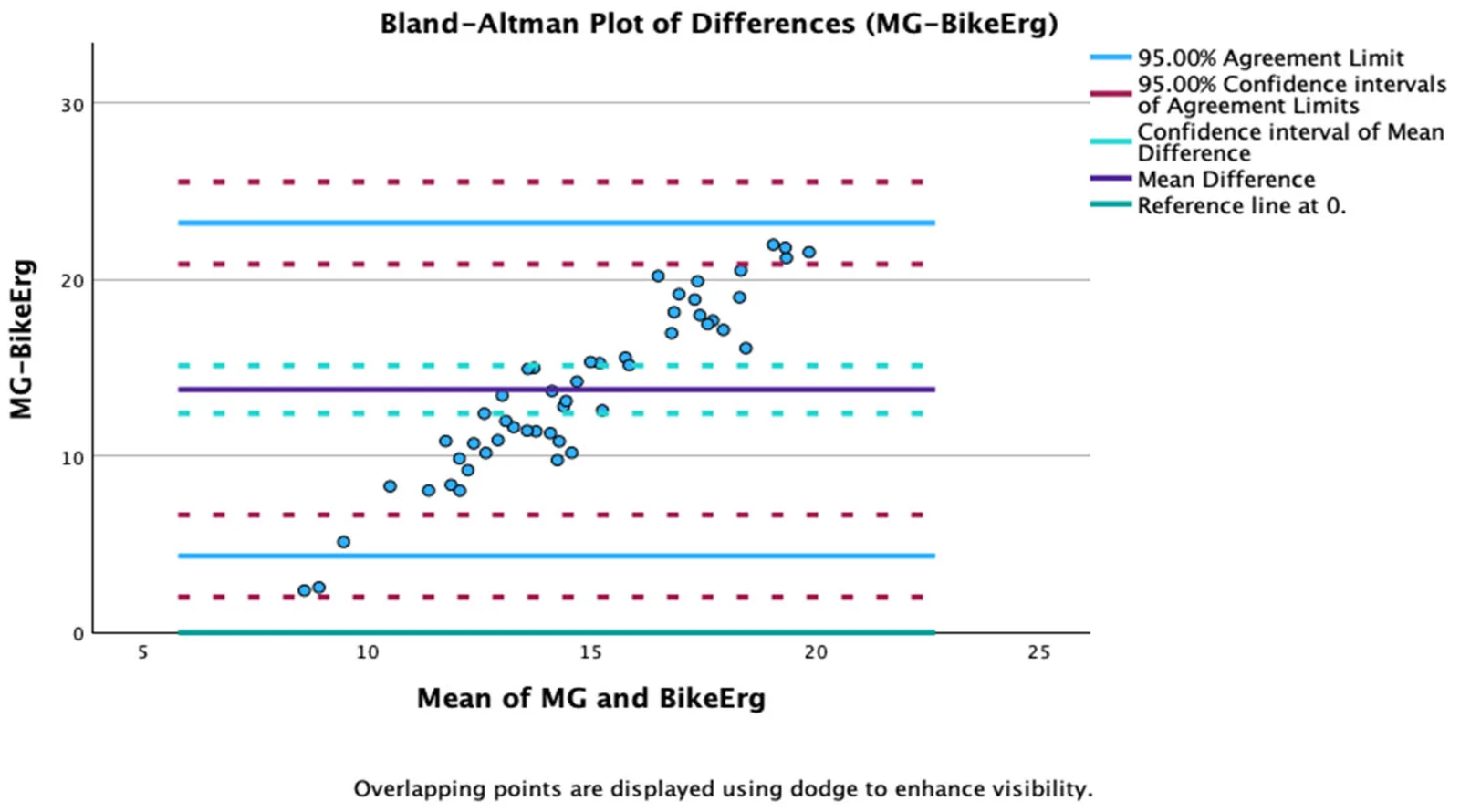
Bland–Altman plot illustrating agreement between MG estimates and BikeErg measurements. The solid grey line indicates the mean difference; the dotted turquoise lines indicate the 95% confidence interval for the mean difference. The solid blue lines indicate the 95% limits of agreement, and the dashed red lines indicate the 95% confidence intervals for the upper and lower limits of agreement.

**Figure 9:**
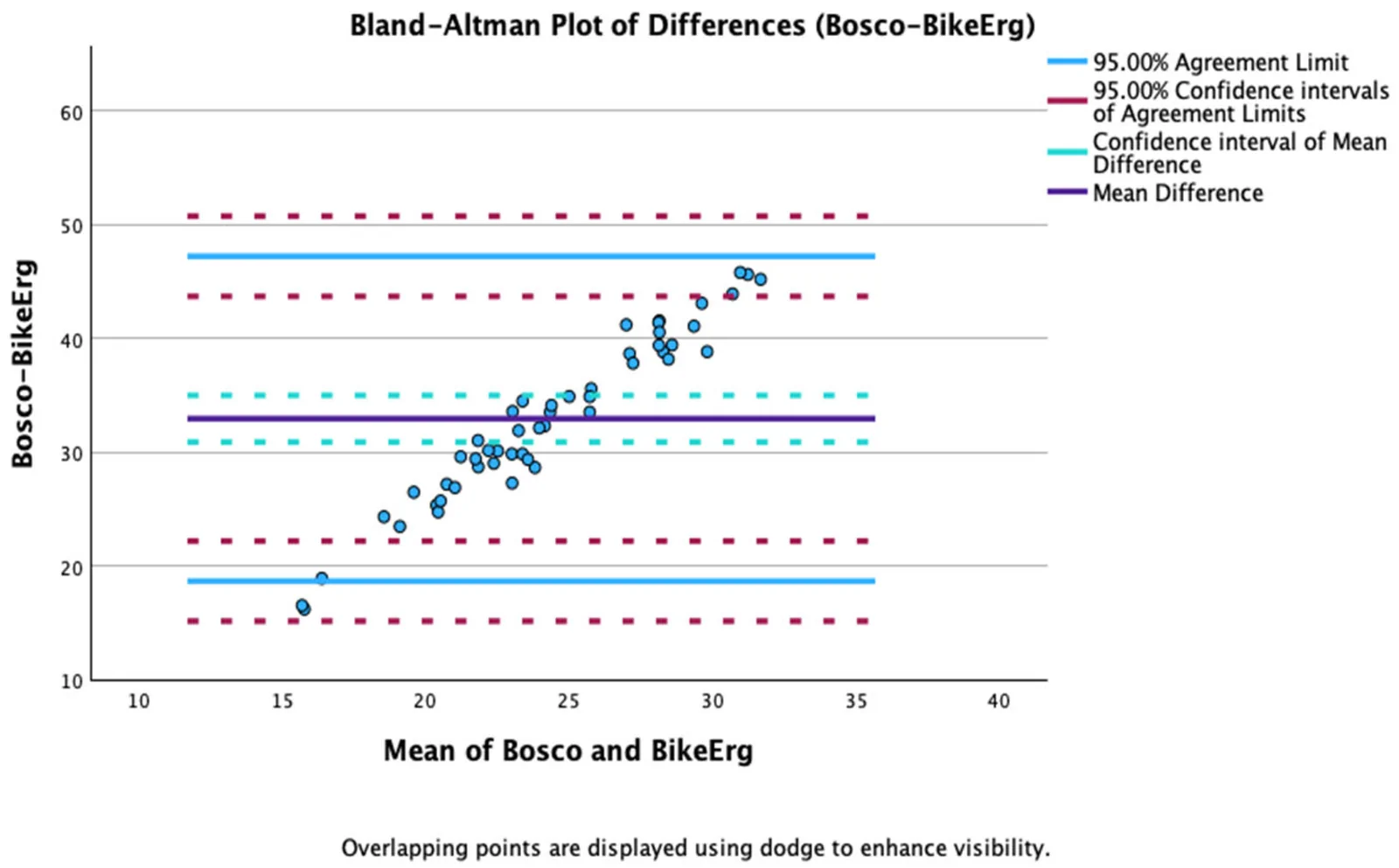
Bland–Altman plot illustrating agreement between Bosco estimates and BikeErg measurements. The solid grey line indicates the mean difference; the dotted turquoise lines indicate the 95% confidence interval for the mean difference. The solid blue lines indicate the 95% limits of agreement, and the dashed red lines indicate the 95% confidence intervals for the upper and lower limits of agreement.

**Table 1:** Descriptive statistics of average power output across computational models and BikeErg reference.

Method	M (W/kg)	SD (W/kg)	CV (%)
Bosco	40.79	7.65	18.75
MG	21.60	5.13	23.76
MGM-15	4.20	0.54	12.76
BikeErg	7.83	0.94	12.02

Note. CV = coefficient of variation; N = 51. MG = Miron Georgescu model; MGM-15 = modified MGM for 15 s repeated jump test; BikeErg = Concept2 cycling ergometer.

**Table 2:** One-way repeated-measures ANOVA: effect of method on average power output.

Source	SS	df	MS	*F*	*p*	η^2^p
Method (PU)	42,057.76	1.03	40,997.85	1082.05	<0.001	0.956
Error	1943.44	51.29	37.89			

Note. Degrees of freedom Greenhouse–Geisser-corrected. SS = sum of squares; MS = mean square; η^2^p = partial eta squared.

**Table 3:** Bonferroni-adjusted pairwise comparisons of average power output between methods.

Comparison	Mean Diff. (W/kg)	*SE*	*p*	95% CI
MG vs. Bosco	−19.19	0.36	<0.001	[−20.17, −18.20]
MG vs. MGM-15	+17.40	0.65	<0.001	[+15.63, +19.18]
MG vs. BikeErg	+13.77	0.67	<0.001	[+11.92, +15.62]
Bosco vs. MGM-15	+36.59	1.00	<0.001	[+33.85, +39.33]
Bosco vs. BikeErg	+32.96	1.02	<0.001	[+30.16, +35.76]
MGM-15 vs. BikeErg	−3.63	0.12	<0.001	[−3.95, −3.31]

Note. All comparisons Bonferroni-adjusted. Mean difference = I − J. Positive values indicate I > J.

**Table 4:** Pearson correlations and linear regression coefficients.

Variable	r	*p*	B	SE	β	95% CI for B
MGM-15–Bosco	0.977	<0.001	13.95	0.43	0.977	[13.09, 14.82]
MGM-15–BikeErg	0.473	<0.001	0.83	0.22	0.473	[0.39, 1.28]

Note. Constant for MGM-15–Bosco: B = −17.79 (95% CI: −21.45, −14.12). Constant for MGM-15–BikeErg: B = 4.34 (95% CI: 2.46, 6.22). *r* = Pearson correlation; *B* = unstandardized regression coefficient; β = standardized coefficient.

**Table 5:** Regression of paired differences on the mean of each model and BikeErg (proportional bias test).

Comparison	B	SE	R^2^	F	*p*
MGM-15–BikeErg	−0.73	0.15	0.313	22.30	0.001
Bosco–BikeErg	1.75	0.06	0.952	975.77	0.001
MG–BikeErg	1.63	0.08	0.894	413.79	0.001

Note. B = unstandardized regression coefficient (slope) of the paired difference (Model − BikeErg) regressed on the mean of the two measures; a nonzero slope indicates proportional bias. df = 1, 49 for all models. N = 51.

## Data Availability

Data are contained within the article.

## Abbreviations

The following abbreviations are used in this manuscript:

MG	Miron Georgescu
MGM-15	Modified Miron Georgescu 15 s
W/kg	Watts per kilogram
PU	Average Power
T_f_	Flight time
T_c_	Contact time
g	Gravitational acceleration
h	Jump height
